# Seasonal variation in children with developmental dysplasia of the hip

**DOI:** 10.1007/s11832-014-0558-3

**Published:** 2014-02-06

**Authors:** Randall T. Loder, Cody Shafer

**Affiliations:** Department of Orthopaedic Surgery, Indiana School of Medicine, James Whitcomb Riley Children’s Hospital, Indiana University, ROC 4250, 705 Riley Hospital Drive, Indianapolis, IN 46202 USA

**Keywords:** DDH, Birth month, Season, Cosinor analysis, Variation

## Abstract

**Background:**

It has been postulated that developmental dysplasia of the hip (DDH) is more frequent in infants born in the winter months. It was the purpose of this study to ascertain if there was any seasonal variation in DDH at the author’s institution and compare/contrast our results with those in the literature using rigorous mathematical fitting.

**Methods:**

All children with DDH treated at the author’s institution from 1993 to 2012 were identified. The month of birth was recorded and temporal variation was analyzed using cosinor analysis. Similar data from the literature was analyzed.

**Results:**

There were 424 children (363 girls, 61 boys). An additional 22,936 children were added from the literature for a total of 23,360. Pearson’s Chi-square test demonstrated a non-uniform distribution in the month of birth for both our 424 children as well as the combined literature series in both the Northern and Southern hemispheres. Cosinor analysis of the 424 children demonstrated double peaks in mid-March and mid-October. For the entire 23,360 children, no seasonal variation was observed in 2,205 (9.4 %), a single winter peak in 16,425 (70.3 %), a single summer peak in 1,280 (5.5 %), and double peaks in the spring and autumn in 3,450 (14.8 %).

**Conclusions:**

This study partly supports the hypothesis of tight clothing/cold temperature as one factor in the etiology of DDH with the tighter clothing/swaddling increasing the risk of DDH. However ~20 % of the DDH births demonstrated a non-winter peak. The single summer and double spring/autumn peaks, as well as in those series where no seasonal variation was noted, refutes the cold winter clothing hypothesis. Perhaps these different patterns in seasonal variation represent the heterogeneity of the genetic factors in DDH interacting with external factors (temperature and clothing) and internal factors (metabolic). Further study will be required to understand these different patterns in DDH seasonal variation.

**Level of evidence:**

IV—case series.

## Introduction

There is good evidence that developmental dysplasia of the hip (DDH) is more frequent in children born in the colder months in both the Northern [1–29] and Southern hemispheres [30–33]. The reverse, with peaks in the spring/summer, have also been described [34–37] as well as double peaks [38]. Finally, complete absence of seasonal variation has been noted [39–47]. Can these different patterns be explained and/or related to the etiology of DDH?

There are at least three hypotheses addressing the predominance of winter births in the etiology of DDH. (1) Infants born in colder months demonstrate poorer acetabular development compared to those born in warmer months [48]. The same has been noted in adults needing total joint arthroplasty [49] with an increased prevalence of hip osteoarthritis in patients born in the winter. The reason why is unknown, (2) but may correlate with the second hypothesis. Infants born in colder months often need tighter clothing or swaddling to protect against the cold [50–52] and swaddling is known to increase the risk of DDH [53–55]. (3) Finally, obstetric pelvic insufficiency shows a seasonal variation [56] with a peak in November–December; increased pelvic insufficiency from higher levels of relaxing hormones could be easily transferred to the infant and result in increased DDH.

It was the purpose of this study to further investigate the seasonal variation in birth month in DDH using formal mathematical modeling. We wished to study both the cases from the author’s institution as well as the literature, and compare/contrast the findings.

## Methods

All children with DDH treated at the author’s institution over the 10-year period from 2003 through 2012 were identified by the ICD9 code of 754.3x and appropriate CPT codes (27256, 27257, 27258, 27259, 27146, 27147, 27151, 27156, and 27165). The charts and radiographs were reviewed to confirm the diagnosis. Children with teratologic, neuromuscular and/or syndromic hip dysplasia were excluded. This study was approved by our local Institutional Review Board.

From the medical records the date of birth, gestational age at birth, gender, race, and treatment method (Pavlik harness/abduction bracing, closed reduction/casting, or open reduction) were collected. We first determined if there was a non-uniform distribution in the month of birth by the Pearson’s Chi-square test. Temporal variation was further analyzed with cosinor analysis [57, 58] which represents a mathematical best fit of the data to a curve defined by the equation *F*(*t*) = *M* + *A* cos(*ωt* + *ϕ*), where *M* is the mean level (termed mesor), *A* the amplitude of the cosine curve, *ϕ* the acrophase (phase angle of the maximum value), *ω* the frequency (which for monthly analysis is 360°/12 = 30°), and *t* is time (which in this case is each month). The overall *p* and *r*^2^ value distribution is given for the rhythmic pattern described by the cosinor equation for *M*, *A*, and *ϕ*. The data was analyzed for the entire period of 12 months as well as decreasing increments of one month. A best monthly fit may not be over a period of 12 months, but a different time span (e.g., seven or six months periodicity). Cosinor analyses were performed with ChronoLab 3.0™ software (see “Acknowledgments”). For all analyses, a *p* < 0.05 was considered statistically significant.

Data from the literature was also extracted and subjected to cosinor analysis. Studies not published in English were translated with translate.google.com. The search was the same one used in a previous study by the senior author [53], which was a systematic review of articles on DDH in infants focusing on etiology, epidemiology, and diagnosis. Exclusion criteria were those manuscripts discussing surgery, therapy, rehabilitation or not having an English abstract/summary. There were certain difficulties in searching the literature on this topic because of the many variant names for DDH. The most commonly used modern terms are “developmental dysplasia of the hip” or DDH; and “congenital hip dislocation” or CDH. Archaic terms include “congenital dislocation” or “congenital hip” or “congenital subluxation of the hip” or “congenital dysplasia of the hip.” Even with controlled vocabularies, each database uses a different subject term, e.g., Medline’s (Medical Subject Headings or MESH) heading is “Hip Dislocation, Congenital”; EMBASE uses “Congenital Hip Dislocation”, Web of Science uses “Congenital Dislocation”, and the historical Index-Catalogue uses “Hip Joint, Dislocation of, Congenital.”

The databases used in this review were PubMed Medline (1947–2010) (http://www.ncbi.nlm.nih.gov/pubmed/), Ovid Medline^®^ (1947–2010), EMBASE (1987–2010), WorldCat (1880–2010) (books and theses) (http://firstsearch.oclc.org/), Web of Knowledge (1987–2010), and IndexCat [Index Catalogue of the Library of the Surgeon-General’s Office (1880–1961)] (http://www.indexcat.nlm.nih.gov/). Individual orthopedic journals were also searched for articles published prior to 1966 that predate electronic indexing, including *Journal of Bone and Joint Surgery* (*American and British*)*, Clinical Orthopaedics and Related Research*, and *Acta Orthopaedica Scandinavica*. Hand searching and citation searching were also performed. Google Scholar (1880–2010) (http://scholar.google.com/) was searched as a final check, but we did not find any additional articles. Age groups were limited to those <18 years old; duplicate citations were removed.

This search resulted in 2,277 unique manuscripts which were reviewed to find those that discussed any of the topics regarding DDH and epidemiology, etiology, demographics, incidence, prevalence, race, gender, family history, inheritance, genetics, age, bone age, weight (either birth weight or normal weight), height, growth, maturation, any other anthropometric characteristics, seasonal variation, hormone, endocrine, congenital anomalies, perinatal factors, swaddling, collagen, and opposite hip. Of these 2,277 manuscripts, 422 provided demographic information, with 49 mentioning seasonal variation [1–27, 30, 31, 33–47, 59–63]. Detailed review of these 49 manuscripts resulted in 27 that gave the month of birth or could be extracted from graphical presentation. The remaining 22 studies either mentioned seasonal variation but did not give the data, or only gave it by the various seasons, not by month.

The latitude, average monthly temperature, and average monthly precipitation were ascertained for all locations. The source for the latitude was the National Geographic Atlas of the World [64] and for the average monthly temperature and the World Meteorological Organization, United Nations Statistics Division and the National Oceanic and Atmospheric Administration, Geographic Information Systems, National Climate Data Center [65, 66].

## Results

There were 424 children (363 girls, 61 boys) meeting the study’s inclusion criteria. The majority (340) were Caucasian. There were 281 unilateral cases and 140 bilateral cases. The treatment was a Pavlik harness/abduction orthosis in 283, and operative (closed or open reduction with/without osteotomy) in 126 children. The raw data from our patients and the literature is shown in Table 1. There were a total of 23,360 children with DDH.

**Table 1:** Month of birth data for 23,360 children with DDH

Study	Location	Latitude^a^	Years	*n*	Jan	Feb	Mar	Apr	May	June	July	Aug	Sep	Oct	Nov	Dec
Cyvin [68]	Trondheim, Norway	63	1963–1974	548	31	47	55	45	50	40	41	46	60	61	31	41
Heikkilä [36]	Uusimaa, Finland	60	1966–1975	957	64	64	83	105	96	93	103	65	79	84	65	56
Bjerkreim and van der Hagen [7]	Oslo, Norway	60	1960–1970	1,183	83	101	91	119	108	88	109	85	110	109	87	93
Andrén and Palmén [9]	Sweden	59	1945–1960	1,313	106	117	118	97	100	99	113	107	127	114	120	95
Anand et al. [27]	East England	52	1979–1986	154	24	23	34	31	10	6	10	11	0	5	0	0
Edwards and Record [20, 21]	Birmingham, England	52	1942–1956	186	20	21	22	8	16	15	11	10	12	14	13	24
Schmidt-Peter [14]	Berlin, Germany	52	1950–1960	793	84	59	72	64	46	55	60	62	72	76	66	77
Vencálková and Janata [43]	Liberic, Czechoslovakia	51	1984–1991	453	44	35	36	44	38	28	32	41	44	36	39	36
Uibe [13]	Leipzig, Germany	51	1928–1957	4,345	463	376	423	299	320	286	285	374	364	408	355	392
Gladisch and Scippan [12]	Leipzig, Germany	51	1946–1958	2,958	257	203	246	245	231	206	244	218	284	283	270	271
Wilkinson [35]	Southampton, England	51	1968–1969	23	1	4	2	2	3	1	1	3	4	0	1	1
Kosek [24]	Děčín and Česká Lípa, Czechoslovakia	51	1964–1970	1,048	117	97	85	65	62	71	69	67	107	99	106	103
Zacharias [29]	Karl-Marx-Stadt, Germany	51	1950–1959	553	53	46	43	42	40	44	34	43	49	55	50	54
Tomás [38]	Bardejov, Slovakia	49	1984–1988	1,142	93	111	100	70	72	100	98	114	102	100	98	82
Czéizel et al. [23]	Budapest, Hungary	48	1962–1967	3,000	308	254	228	219	219	214	242	242	243	277	259	295
Illyés [32]	Nyíreghá, Hungary	48	8-year span before 1968	765	75	64	70	49	50	40	38	64	92	71	77	75
Woolf et al. [18]	Utah, USA	41	1951–1961	476	48	30	31	41	38	23	32	40	47	52	45	49
Present study	Indiana, USA	40	1993–2012	424	23	46	46	42	22	21	32	32	40	45	39	36
Robinson [17]	New York City	40	1955–1963	339	17	41	38	20	23	19	22	27	34	30	33	35
Valdivieso Garcia et al. [34]	Córdoba, Spain	38	1981–1984	323	22	25	36	34	32	39	26	23	17	20	23	26
Nagura [137]	Tokyo, Japan	36	1927–1941	1,306	286	156	129	88	62	42	49	63	78	90	144	119
Haginomori [2]	Kōchi, Japan	34	1961–1963	106	23	15	14	10	7	0	1	0	3	9	7	17
Chen et al. [25]	Tel Aviv, Israel	32	1962–1967	84	7	9	9	8	2	4	7	1	3	11	11	12
Medalie et al. [10]	Jerusalem, Israel	31	1954–1960	313	31	18	28	15	17	16	21	27	26	37	35	42
Aguirre-Negrete et al. [28]	Guadalajara, Mexico	21	1985–1986	127	8	9	14	3	6	4	7	10	2	17	28	19
Charlton [33]	South Australia	−35	1947–1962	145	10	10	8	14	17	16	13	17	9	15	9	7
Cohen [31]	Victoria, Australia	−39	1961–1965	230	10	22	25	21	33	27	8	16	18	17	20	13
Dykes [30]	Southland, New Zealand	−46	1958–1967	66	3	3	6	6	9	12	4	8	4	3	4	4
Total				23,360												
No seasonal variation [7, 17, 31, 43]				2,205												
Seasonal variation				21,155												
Summer peaks [34, 36]				1,280												
Bimodal peaks [9, 35, 38, 68]				3,450												
Winter peaks [2, 10, 12, 13, 18, 20, 21, 23–25, 27–30, 32, 33, 137]				16,425												

^a^In degrees; Northern hemisphere values are denoted as positive and Southern hemisphere as negative

**Fig. 1:**
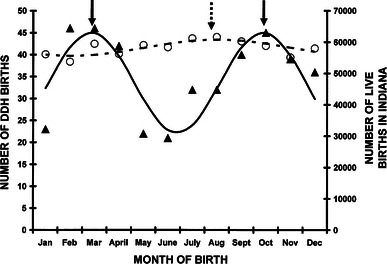
Month of birth for 424 children with developmental dysplasia of the hip. Cosinor analysis demonstrated an excellent fit using a 7-month periodicity with the equation: number of DDH births = 33.61 + 11.45(cos(51.4*t* − 25.7) − 122), where *t* = 1 is January, 2 is February, 3 is March, etc. This was statistically significant (*r*^2^ = 0.70, *p* = 0.005). The peaks are March 13 and October 12 (*solid arrows*). The data points are the *black triangles* and the best fit represented by the *bold black line*. The numbers of births in the state of Indiana are represented by the *open circles* and the 12-month cosinor fit by the *hatched line* represented by the equation: number of live births = 58,234 + 2,586(cos(30*t* − 15) − 208), where *t* = 1 is January, 2 = February, 11 = November, 12 = December. This was statistically significant (*r*^2^ = 0.60, *p* = 0.017). The peak was July 30 (*hatched arrow*)

In our 424 children, there was a non-uniform distribution in the month of birth (Pearson’s *χ*^2^ = 27.13, *df* = 11, *p* = 0.0044). Cosinor analysis of our data demonstrated double peaks in the month of birth (Fig. 1; Table 2). The peak was mid-March and mid-October. As known, DDH can vary from mild hip subluxation to complete, fixed, non-reducible dislocations. The data in the charts did not always give ample information to determine the exact severity of DDH. However, a proxy of the severity is the method of treatment needed. Those only needing a Pavlik harness are likely Ortolani or Barlow positive hips that are stable in the harness; those needing a closed reduction and casting represent those with a dislocated but reducible hip; and those needing formal open reduction are the fixed, non-reducible or unstable dislocations. Despite these differences in severity of the DDH, all of these groups demonstrated seasonal variation (Table 2). The peaks were nearly always mid-March and mid-October except for children treated operatively who demonstrated a single peak in mid-October. The data for live births in our state during 2000–2010 [67] demonstrated a single mild peak in late July.

**Table 2:** Cosinor analyses of DDH births in Indiana

	*n*	Periodicity	*r* ^2^	*p* value	*M*	*A*	*ϕ*	Month
All	424	7	0.70	0.005	33.61	11.45	122	Mar 13, Oct 12
VD	204	7	0.75	0.002	16.11	5.88	125	Mar 15, Oct 14
CS	183	7	0.69	0.005	14.38	5.86	117	Mar 10, Oct 9
Breech	125	8	0.57	0.023	9.92	3.19	44	Jan 30, Sep 30
Vertex	259	7	0.86	<0.001	20.23	9.00	132	Mar 19, Oct 18
Unilateral	281	7	0.71	0.004	22.36	7.05	118	Mar 11, Oct 10
Bilateral	140	8	0.43	0.079	10.74	4.30	100	Mar 9, Nov 7
Left	208	6	0.67	0.007	17.34	4.17	174	Mar 29, Sep 28
Right	72	7	0.54	0.032	5.61	2.72	110	Mar 6, Oct 5
Pavlik	260	7	0.61	0.014	20.39	8.46	132	Mar 19, Oct 18
Operative	126	10	0.56	0.025	10.30	2.16	340	Oct 15
Live births in Indiana	698,390	11	0.60	0.017	58,417.00	505.10	226	29-July

*VD* vaginal delivery, *CS* cesarean section, *M* mesor, *A* amplitude, *ϕ* acrophase, *Month* acrophase of cosinor analysis converted to month

**Fig. 2:**
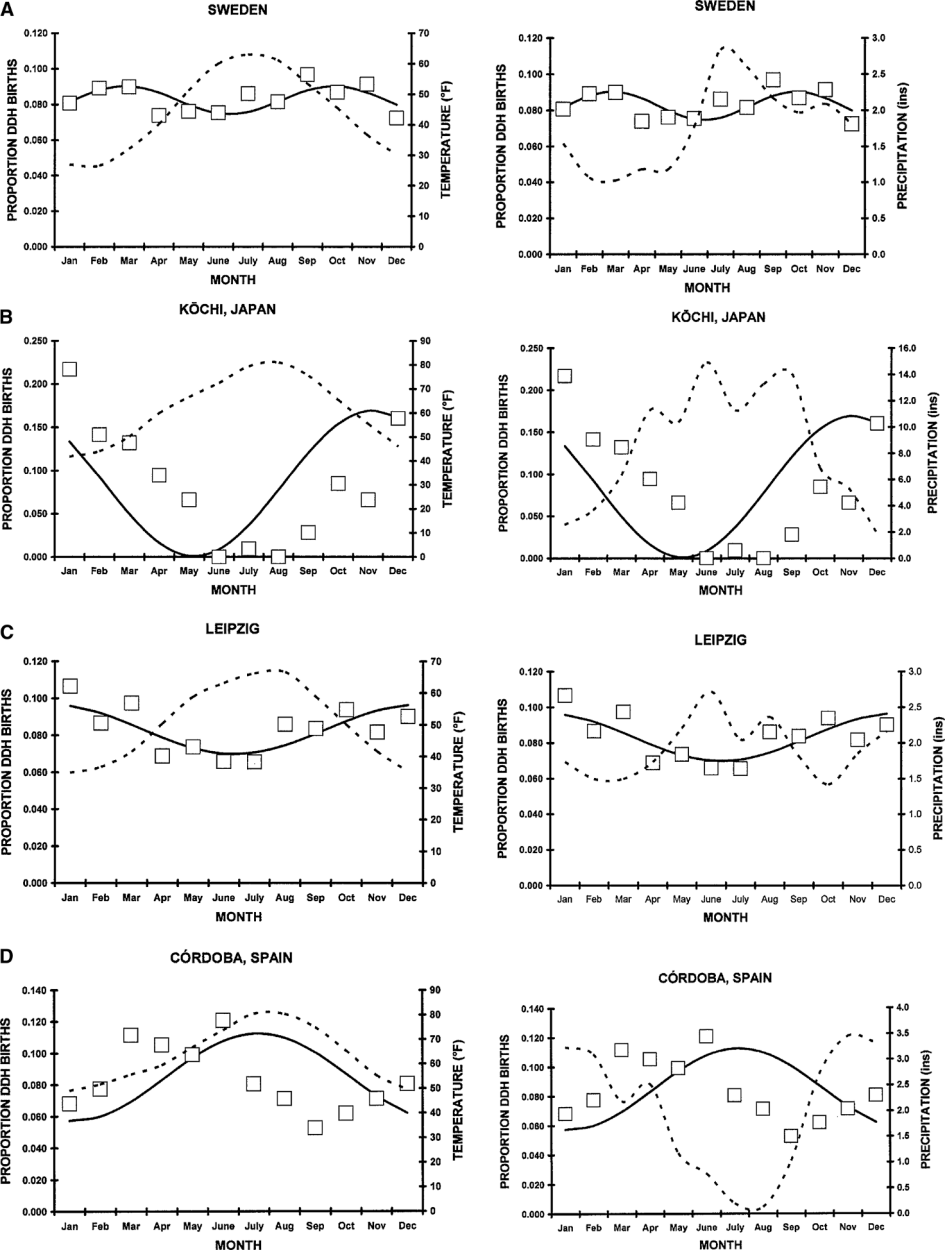
The three different patterns of DDH birth month. The month of birth data is represented by the *open square*; the cosinor fit by the *solid black line*; the average monthly temperature (°F) and precipitation (in.) are shown by the *dotted lines*. **a** A double peak in Sweden [9]. This double fit with a period of seven months was statistically significant and represented by the equation: proportion DDH births = 0.0824 + 0.00786(cos(51.40*t* − 15) − 122). This was statistically significant (*r*^2^ = 0.53, *p* = 0.034). The two peaks were February 20 and September 21. The seasonal variation was small, with a mesor/amplitude ratio of 0.10. **b** A single winter peak seen in Kōchi, Japan [2]. This was statistically significant (*r*^2^ = 0.843, *p* = 0.002) and represented by the equation: proportion DDH births = 0.0853 + 0.00842(cos(30*t* − 15) − 25); the peak was January 26. Note the large seasonal variation, with a mesor/amplitude ratio of 0.99. **c** A single winter peak seen in Leipzig, Germany [13]. This was statistically significant (*r*^2^ = 0.58, *p* = 0.021) and represented by the equation: proportion DDH births = 0.0833 + 0.0133(cos(30*t* − 15) − 350); the peak was December 21. Note the small seasonal variation, with a mesor/amplitude ratio of 0.16. **d** A single summer peak seen in Córdoba, Spain [34]. This was statistically significant (*r*^2^ = 0.84, *p* < 0.001) and represented by the equation: proportion DDH births = 0.0849 + 0.0276(cos(30*t* − 15) − 146); the peak was May 28. Note the moderate seasonal variation, with a mesor/amplitude ratio of 0.33

To analyze for variation in month of birth in the literature data, we first separated the studies by Northern or Southern hemisphere, due to the 6-month differences in seasons. There was a non-uniform distribution in the month of birth for both those in the Northern hemisphere (Pearson’s *χ*^2^ = 252.1, *df* = 11, *p* < 0.0001) and Southern hemisphere (Pearson’s *χ*^2^ = 38.3, *df* = 11, *p* = 0.0001). The results of the cosinor analyses are shown in Table 3. Double peaks similar to ours were noted in Trondheim, Norway [68], Southampton, England [35], Sweden [9] (Fig. 2a), and Bardejov, Slovakia [38]. Most other studies demonstrated a single peak in the winter months (Fig. 2b, c) except in Córdoba, Spain (Fig. 2d) [34] and Uusimaa, Finland [36] which had a single summer peak. There were no statistically significant cosinor fits for those in New York City [17]; Victoria, Australia [31]; Oslo, Norway [7], and Liberic, Czechoslovakia [43]. Of the total 23,360 children with DDH, no seasonal variation was observed in 2,205 (9.4 %), while a seasonal variation was observed in the remaining 21,115 (90.6 %). For these 21,115, the variation was single winter peak in 16,425 (77.6 %), a single summer peak in 1,280 (6.1 %), and double peaks in the spring and autumn in 3,450 (16.3 %).

**Table 3:** Cosinor analyses for month of birth for children with DDH—literature series

Study	Location	Latitude^a^	Years	*n*	Periodicity (months)	*r* ^2^	*p* value	*M*	*A*	*ϕ*	Month
Cyvin [68]	Trondheim, Norway	63	1963–1974	548	6	0.57	0.022	45.68	10.03	167	25-Mar, 24-Sep
Heikkilä [36]	Uusimaa, Finland	60	1966–1975	957	12	0.64	0.01	79.81	18.33	155	6-June
Andrén and Palmén [9]	Sweden	59	1945–1960	1,313	7	0.53	0.034	108.23	10.32	86	20-Feb, 21-Sep
Anand et al. [27]	East England	52	1979–1986	154	12	0.70	0.027	15.96	11.79	56	26-Feb
Edwards and Record [20, 21]	Birmingham, England	52	1942–1956	186	12	0.53	0.034	15.48	5.07	26	27-Jan
Schmidt-Peter [14]	Berlin, Germany	52	1950–1960	793	12	0.58	0.021	66.05	10.91	334	5-Dec
Uibe [13]	Leipzig, Germany	51	1928–1957	4,345	12	0.58	0.02	361.85	57.99	350	21-Dec
Gladisch and Scippan [12]	Leipzig, Germany	51	1946–1958	2,958	12	0.47	0.059	246.48	25.89	306	6-Nov
Wilkinson [35]	Southampton, England	51	1968–1969	23	6	0.50	0.062	2.14	1.14	145	15-Mar, 13-Sep
Kosek [24]	Děčín and Česká Lípa, Czechoslovakia	51	1964–1970	1,048	12	0.83	<0.001	87.26	24.25	335	6-Dec
Zacharias [29]	Karl-Marx-Stadt, Germany	51	1950–1959	553	12	0.75	0.002	46.06	7.38	331	2-Dec
Tomás [38]	Bardejov, Slovakia	49	1984–1988	1,142	7	0.55	0.028	95.08	13.37	39	23-Jan, 24-Aug
Czéizel et al. [23]	Budapest, Hungary	48	1962–1967	3,000	12	0.74	0.002	249.90	35.24	333	4-Dec
Illyés [32]	Nyíreghá, Hungary	48	8 years	765	12	0.66	0.008	63.71	17.89	324	20-Nov
Woolf et al. [18]	Utah, USA	41	1951–1961	476	12	0.56	0.025	39.65	9.18	311	11-Nov
Present study	Indiana	40	1993–2012	424	7	0.70	0.005	33.61	11.45	122	13-Mar, 12-Oct
Valdivieso Garcia et al. [34]	Córdoba, Spain	38	1981–1984	323	12	0.84	<0.001	27.43	8.93	146	28-May
Nagura [137]	Tokyo, Japan	36	1927–1941	1,306	12	0.68	0.006	108.49	75.00	11	11-Jan
Haginomori [2]	Kōchi, Japan	34	1961–1963	106	12	0.84	0.002	9.04	8.93	25	26-Jan
Chen et al. [25]	Tel Aviv, Israel	32	1962–1967	84	12	0.54	0.032	6.98	3.68	359	30-Dec
Medalie et al. [10]	Jerusalem, Israel	31	1954–1960	313	12	0.77	0.001	26.06	10.6	317	17-Nov
Aguirre-Negrete et al. [28]	Guadalajara, Mexico	21	1985–1986	127	12	0.48	0.054	10.56	7.17	329	30-Nov
Charlton [33]	South Australia	−35	1947–1962	145	12	0.56	0.025	12.10	3.70	178	30-June
Dykes [30]	Southland, New Zealand	−46	1958–1967	66	12	0.56	0.024	5.51	2.89	156	7-June

*M* mesor, *A* amplitude, *ϕ* acrophase, *Month* acrophase of cosinor analysis converted to month

^a^In degrees; Northern hemisphere values are denoted as positive and Southern hemisphere as negative

The average monthly temperature and precipitation were overlaid onto the monthly distribution of DDH births. Visual review demonstrated no correlation with average monthly precipitation. For those demonstrating single peaks in DDH births, there was either a positive correlation [increased DDH births with increasing temperature (summer months)], or a negative correlation [increased DDH births with decreasing temperatures (winter months), which conversely indicates increased DDH births in the winter months]. For those with double peaks there was no correlation with either average monthly temperature or precipitation. Thus, the three major patterns of seasonal variation in DDH births cannot be explained simply by average monthly temperature or precipitation.

## Discussion

There are certain limitations to this study. Regarding our own 424 cases, is such a sample large enough to say there is or is not a seasonal variation? We believe it is, since the Pearson Chi-square test demonstrated a non-uniform distribution at a *p* = 0.0044, and cosinor analysis demonstrated an excellent bimodal fit with and *r*^2^ = 0.70 and *p* = 0.005. Next, the definitions of DDH used in the various literature series was likely different from study to study, which might introduce some bias. However the overall Pearson Chi-square test for non-uniform distribution was highly significant for both the Northern hemisphere (*p* < 0.0001), and Southern hemisphere (*p* = 0.0001). There could also be selection bias from the literature review, but it was exhaustive, and all the studies that mentioned seasonal variation were completely reviewed (not just the abstract), and the data extracted where given. Thus, there was minimal selection bias regarding this aspect of the study. Therefore, we believe the data is very representative as both our data as well as that from the literature demonstrate seasonal variation in the month of birth.

This is the first study to mathematically model birth month in children with DDH. Three major patterns were noted: a single peak in the winter, a single peak in the summer, and double peaks in the spring and autumn. In some instances there were no seasonal variations. For those with seasonal variation, a wide spread in the magnitude of the monthly variation, the proportion of the amplitude (*A*) to the mesor (*M*), was noted and varied from 11 % in Leipzig, Germany [12] to 99 % in Kōchi, Japan [2]) (Fig. 2).

This study partially supports the cold winter hypothesis where infants born in the colder months need tighter clothing or increased swaddling to protect the baby from the cold which increases the incidence of DDH. But is this cause or effect? Swaddling is a well known factor in the etiology of DDH [53–55] and is well-demonstrated by two different peoples, the Sámi and Inuit, who both live in the cold circumpolar North but have markedly different incidences of DDH [69–73]. In the Swedish Sámi, the cradleboard (komse) accounts for a high incidence of DDH (24.6 per 1,000) [71]. The Inuit mothers carry their young in a hood inside their parkas (amauti) which abducts the hips around their backs; they have an incidence of DDH similar to Caucasians [70]. Also, once changes in swaddling during winter months were made in Japan, the incidence of DDH dropped markedly [50, 52]. However, the single summer and double spring/autumn peaks, as well as those with no seasonal variation contradict the cold winter clothing hypothesis. Thus, there must be other factors involved and likely represents the interaction between genetics, external factors (temperature, clothing) and internal factors (metabolic).

Collagen metabolism is altered in DDH [74–78] with increased joint laxity [79–85]. Relaxin, which stimulates collagenase, alters the connective tissue and could potentially lead to the development of DDH. At six weeks postpartum, relaxin levels can no longer be measured in the sera of mothers but can still be measured in their milk [86]; it is possible that these high relaxin concentrations transmitted to the child via breast milk might lead to DDH.

Obstetric pelvic insufficiency [56] has a seasonal variation with a peak in November–December. Women with pelvic insufficiency in the third trimester have higher serum relaxin levels compared to those without [87] and a 3-fold [88] to 7-fold [89, 90] increase in children with DDH. This is possibly due to more relaxin transferred to the infant resulting in DDH. Others have noted the reverse, where the incidence of DDH is increased in those with low relaxin levels [91], likely due to a longer labor or passage through a tighter birth canal. Also, there is no correlation between serum relaxin in umbilical cord blood and neonatal hip instability [91, 92] and no seasonal variation in relaxin levels has been discovered. Thus relaxin is not the entire picture.

Other possible internal factors are seasonal variations in the hormonal/endocrine milieu, nutritional issues, and viral illnesses. DDH occurs predominantly in females and a hormonal/endocrine relationship has long been suspected. Estrogen and its metabolites [93–96] as well as estrogen receptors [97, 98] demonstrate abnormalities in children with DDH. Estrogen is known to influence ligamentous laxity; anterior cruciate ligament injuries are more frequent in women athletes in their midcycle ovulatory phase when both estrogen and luteinizing hormone levels peak [99]. Seasonal variation has been noted in dehydroepiandrosterone sulfate levels in Denmark, with two peaks, one in September and one in March [100]. Estradiol levels peak in early summer in Oslo (59°56′N) and early winter in Tromsø (69°42′N) Norway [101]. High fat/low fiber diets result in elevated serum estrogens [102, 103] with seasonal variation in estrogen levels [104]. Thus, some of the seasonal variation in DDH could be explained by seasonal variations in estrogens. Progesterone has also been implicated in DDH [105]. In Ontario, Canada, progesterone (as well as melatonin) levels during the autumn and winter (dark months) were significantly higher than during the spring and summer (light months) in follicular fluid and the opposite with estradiol [106].

Another hormone to consider is vitamin D [107] which is well known to have a seasonal variation with peak levels in the summer [108–114]. It has also been implicated in DDH. Homozygosity for the mutant Taq1 vitamin D receptor *t* allele is associated with an increased acetabular index [97]. High vitamin D levels reduce progesterone and estradiol levels [115]. Low maternal levels of vitamin D result in small for gestational age infants [116] and increased levels results in heavy infants [117]. Heavy, large infants have an increased incidence of DDH [15, 44, 118–120]. Thus, high vitamin D levels in the summer with its lower estrogen levels, supports the winter predominance of DDH. However, low levels of vitamin D in small for gestational age babies should demonstrate an increase in DDH, which is contrary to our present understanding [15, 44, 118]. In heavier, larger infants, increased vitamin D levels with lower estrogen levels should demonstrate a lower incidence of DDH, but the opposite occurs [119–121]. Vitamin D levels in Caucasians which decrease with increasing latitude [122] and less sun exposure are also modulated by genetic factors. The genetic variability for vitamin D levels ranges from 14 % to 70 % [112, 113, 123–125]. In Almeria, Spain (36°N) vitamin D levels during pregnancy were highest in the summer and lowest in the winter [126]; and regardless of season, increased with increasing gestational age. In Japan, people living in coastal areas demonstrated lower vitamin D levels [127].

Other factors to consider are vitamin E and A, where a deficiency leads to muscle weakness/myopathy [107]. Muscle weakness around the hip could theoretically result in hip instability. Levels of vitamin A in Japan [128] and Spain [129] are lower in the winter than summer and in France [130] lower in the winter than the autumn. This could also possibly explain the increased incidence of DDH in the winter months. Vitamin C levels in France are higher in the winter and spring [130]. Vitamin C is important in collagen synthesis/metabolism. Perhaps the higher winter/spring vitamin C level, with potentially better collagen explains the increased incidence of DDH in those with summer peaks.

Melatonin levels in infants demonstrate seasonal variation. In Tel Aviv, Israel, melatonin levels in 8-week-old infants were highest in June and lowest in December [131]. In Adelaide, Australia, there was no difference between summer and winter melatonin levels in adults [132]. In Norway, low levels were seen in early summer in both Oslo (59°56′N) and Tromsø (69°42′N); in Tromsø there was a single peak in the late summer, while in Oslo there were two peaks, one in late winter and another in late summer [101]. Unfortunately, there have been no investigations regarding melatonin and DDH. However, the marked differences in seasonal variation between these different studies may help explain the variability seen in this study, since melatonin levels are also involved with reproductive/sex hormone levels [133, 134].

Maternal viral illness has also been implicated in the etiology of DDH [32]; enterovirus is a common viral infection that can result in dehydration, and, thus, possibly mild oligohydramnios. The enterovirus peak in temperate climates is in the summer and early fall ([135], Welch, 2003 #1124 [136]). This might explain the summer and autumn peaks seen in this study.

One final interesting finding was noted. Most of the series in this study are from high latitudes; 51.9 % of the cases in this series were from locations having a latitude ≥50°, and 87.9 from locations ≥40°. Does this indicate that DDH is more common in higher latitudes, for many different reasons? Rather does it simply reflect the ethnicities of those peoples living in different latitudes who also have different genetic tendencies for DDH? Much work remains to explain these different patterns in the seasonal variation of DDH and the complex interaction of the various extrinsic physical (clothing/temperature/exposures), intrinsic physical (breech position, oligohydramnios, birth order), metabolic (hormonal), and genetic factors in children with DDH.

## References

[1] Nagura S (1955). Zur ätiologie der angeborenen hüftverrenkung (The aetiology of congenital dislocation of the hip). Zentralbl Chirur.

[2] Haginomori K (1966). A statistical study of congenital dislocation of the hip in the south-western part of Kochi prefecture. Shikoku Acta Medica.

[3] Watanabe M, Saito A, Takeda K, Ando M (1993). Incidence of congenital dislocation of the hip (CDH) before and after preventive management—an analysis from newborn CDH and late diagnosed cases. Prevention of congenital dislocation of the hip in infants: experience and results in Japan.

[4] Wada N, Kato N, Mori S, Abe H, Hirano N, Ando M (1993). Evaluation of screening for congenital dislocation of the hip in Tokushima Prefecture. Prevention of congenital dislocation of the hip in infants: experience and results in Japan.

[5] Iwasaki K, Takahashi K, Ando M (1993). Annual changes in the incidence of congenital dislocation of the hip in infants and the rate of successful reduction in Nagasaki city. Prevention of congenital dislocation of the hip in infants: experience and results in Japan.

[6] Walker JM (1977). Congenital hip disease in a Cree-Ojibwa population: a retrospective study. Can Med Assoc J.

[7] Bjerkreim I, van der Hagen CB (1974). Congenital dislocation of the hip in Norway. V. Evaluation of genetic and environmental factors. Clin Genet.

[8] Andrén L (1962). Pelvic instability in newborns with special reference to congenital dislocation of the hip and hormonal factors. A roentgenologic study. Beitr Orthop Traumatol.

[9] Andrén L, Palmén K (1963). Seasonal variation of birth dates of infants with congenital dislocation of the hip. Acta Orthop Scand.

[10] Medalie JH, Makin M, Alkalay E, Yofe J, Cochavi Z, Ehrlich D (1966). Congenital dislocation of the hip. A clinical–epidemiological study, Jerusalem 1954 to 1960. I. Retrospective incidence study. Isr J Med Sci.

[11] Díaz AF, Navas LS, Viladrich RA (1997). Factores obstétricos y perinatales en la luxación congénita de cardera (Obstetrical and perinatal risk factors for congenital dislocation of the hip). An Esp Ped.

[12] Gladisch M, Scippan R (1964). Über die jahreszeitliche verteilund der hüftdysplasie (Seasonal distribution of dysplasia of the hip). Beitr Orthop Traumatol.

[13] Uibe P (1959). Einfluß der jahreszeiten auf die häufigkeit der luxationshüften (Seasonal influences on incidence of hip luxations). Zentralbl Chirur.

[14] Schmidt-Peter P (1962). Noch einmal: einfluß der jahreszeiten auf die häufigkeit der luxationshüfte. Beitr Orthop Traumatol.

[15] Patterson CC, Kernohan WG, Mollan RAB, Haugh PE, Trainor BP (1995). High incidence of congenital dislocation of the hip in Northern Ireland. Paediatr Perinat Epidemiol.

[16] Wynne-Davies R (1970). A family study of neonatal and late diagnosis congenital dislocation of the hip. J Med Genet.

[17] Robinson GW (1968). Birth characteristics of children with congenital dislocation of the hip. Am J Epidemiol.

[18] Woolf CM, Koehn JH, Coleman SS (1968). Congenital hip disease in Utah: the influence of genetic and nongenetic factors. Am J Hum Genet.

[19] Slater BCS, Watson GI, McDonald JC (1964). Seasonal variation in congenital abnormalities. Preliminary report of a survey conducted by the Research Committee of Council of the College of General Practitioners. Br J Prev Soc Med.

[20] Edwards JH (1961). Seasonal incidence of congenital disease in Birmingham. Ann Hum Genet.

[21] Record RG, Edwards JH (1958). Environmental influences related to the aetiology of congenital dislocation of the hip. Brit J Prev Soc Med.

[22] Pap K (1956). Einfluß der jahreszeiten aui die haüfigkeit der angeborenen hüftverrenkung (Effect of seasons of the year on incidence of congenital hip dislocations). Zentralbl Chirur.

[23] Czéizel A, Vizkelety T, Szentpéteri J (1972). Congenital dislocation of the hip in Budapest, Hungary. Br J Prev Soc Med.

[24] Kosek P (1973). Sezónní vykyvy ve frekvenci kycelních dysplazií—príspevek k etiologii (Seasonal occurrence of the frequency of the hip dysplasia—a contribution to its etiology). Acta Chir Orthop Traumatol Cechoslov.

[25] Chen R, Weissman SL, Salama R, Klingberg MA (1970). Congenital dislocation of the hip (CDH) and seasonality: the gestational age of vulnerability to some seasonal factor. Am J Epidemiol.

[26] Weissman SL, Salama R (1966). Treatment of congenital dislocation of the hip in the newborn infant. J Bone Jt Surg [Br].

[27] Anand JK, Moden I, Myles JW (1992). Incidence of neonatal hip instability: are there seasonal variations?. Acta Orthop Belg.

[28] Aguirre-Negrete MG, García de Alba-García JE, Ramírez-Soltero SE (1991). Luxación congénita de cadera y estacionalidad (Congenital hip dislocation and the seasons). Bol Méd Hosp Inf México.

[29] Zacharias J (1960). Über den einfluß der jahreszeiten auf die luxatonshüfte im gebiet van Karl-Marx-Stadt. Beitr Orthop Traumatol.

[30] Dykes RG (1975). Congenital dislocation of the hip in Southland. N Z Med J.

[31] Cohen P (1971). Seasonal variation of congenital dislocation of the hip. J Interdiscip Cycle Res.

[32] Illyés Z (1968). Die rolle der jahreszeiten beim entstehen der huftluxation (The role of seasons in development of hip dislocation). Beitr Orthop Traumatol.

[33] Charlton PJ (1966). Seasonal variation in incidence of some congenital malformations in two Australian samples. Med J Aust.

[34] Valdivieso Garcia JL, Lopez FB, Losa LMO, Lexcano AR (1989). Incidencia estacional en la luxación congénita de cadera. Un factor de riesgo (Seasonal incidence in congenital dislocation of the hip. A risk factor). An Esp Ped.

[35] Wilkinson JA (1972). A post-natal survey for congenital displacement of the hip. J Bone Jt Surg [Br].

[36] Heikkilä E (1984). Congenital dislocation of the hip in Finland. An epidemiologic analysis of 1035 cases. Acta Orthop Scand.

[37] Klingberg MA, Chen R, Chemke J, Levin S (1976). Rising rates of congenital dislocation of the hip. Lancet.

[38] Tomás V (1989). Vyskyt a liecba vrodenych dysplazii bedroveho klbu v okreese Bardejov az obdobie 5 rokovv 1984–1988 (Incidence and treatment of inborn dysplasia of the hip joint in the region of Bardejov in the period of 1984–1988). Acta Chir Orthop Traumatol Cechoslov.

[39] Rabin DL, Barnett CR, Arnold WD, Freiberger RH, Brooks G (1965). Untreated congenital hip disease. A study of the epidemiology, natural history and social aspects of the disease in a Navajo population. Am J Public Health.

[40] Siffel C, Alverson CJ, Correa A (2005). Analysis of seasonal variation of birth defects in Atlanta. Birth Defects Res (Part A).

[41] Barlow TG (1962). Early diagnosis and treatment of congenital dislocation of the hip. J Bone Jt Surg [Br].

[42] Noble TC, Pullan CR, Craft AW, Leonard MA (1978). Difficulties in diagnosing and managing congenital dislocation of the hip. BMJ.

[43] Vencálková S, Janata J (2009). Souborné zhodnocení screeningu vyvojové dysplazie kychelního kloubu v regionu Liberec za obdobi 1984–2005 (Evaluation of screening for developmental dysplasia of the hip in the Liberec region in 1984–2005). Acta Chir Orthop Traumatol Cechoslov.

[44] Bower C, Stanley FJ, Kricker A (1987). Congenital dislocation of the hip in Western Australia. Clin Orthop.

[45] Phillips LI (1968). Congenital dislocation of the hip in the newborn. A survey at National Women’s Hospital 1954–1968. N Z Med J.

[46] Doig JR, Shannon FT (1975). Congenital dislocation of the hip. An evaluation of neonatal diagnosis. N Z Med J.

[47] Artz TD, Levine DB, Lim WN, Salvati E, Wilson PD (1975). Neonatal diagnosis, treatment and related factors of congenital dislocation of the hip. Clin Orthop.

[48] Mizuno H, Ito A, Hotta I, Ohashi K, Nomura S, Hibino S (1961). Influences of birth-season on development of the infant hip-joint. J Nagoya Med Assoc.

[49] Nagamine S, Sonohata M, Kitajima M, Kawano S, Ogawa K, Mawatari M (2011). Seasonal trends in the incidence of hip osteoarthritis in Japanese patients. Open Orthop J.

[50] Ishida K (1977). Prevention of the development of the typical dislocation of the hip. Clin Orthop.

[51] Ando M (1993). Prevention of congenital dislocation of the hip in infants: experience and results in Japan.

[52] Ishida K, Ando M (1993). Prevention of the onset of congenital dislocation of the hip. Prevention of congenital dislocation of the hip in infants: experience and results in Japan.

[53] Loder RT, Skopelja EN (2011) The epidemiology and demographics of hip dysplasia. ISRN Orthopedics 10.5402/2011/238607 PMC4063216 24977057

[54] Mahan ST, Kasser JR (2008). Does swaddling influence developmental dysplasia of the hip?. Pediatrics.

[55] van Sleuwen BE, Engleberts AC, Boere-Boonekamp MM, Kuis W, Schulpen TWJ, L’Hoir MP (2007). Swaddling: a systematic review. Pediatrics.

[56] Berezin D (1954). Pelvic insufficiency during pregnancy and after paturition. Acta Obstet Gynecol Scand.

[57] Nelson W, Tong YL, Lee J-K, Halberg F (1979). Methods for cosinor-rhythmometry. Chronobiologia.

[58] Faure A, Nemoz C, Claustrat B (1990). A graphical and statistical method for investigation of time series in chronobiology according to the cosinor procedure. Comput Biol Med.

[59] Bjerkreim I (1974). Congenital dislocation of the hip in Norway. I. Late-diagnosis CDH. Acta Orthop Scand Suppl.

[60] Bjerkreim I (1974). Congenital dislocation of the hip in Norway. II: detection of late cases. Acta Orthop Scand Suppl.

[61] Bjerkreim I (1974). Congenital dislocation of the hip in Norway. III. Neonatal CDH. Acta Orthop Scand Suppl.

[62] Bjerkreim I (1974). Congenital dislocation of the hip in Norway. IV. The incidence in southeast Norway. Acta Orthop Scand Suppl.

[63] Bjerkreim I (1976). Congenital dislocation of the hip joint in Norway: a clinical-epidemiologic study. J Oslo City Hosp.

[64] Grosvenor GM, Graves W, English MA, Shupe JF, Dorr JF, Payne OGAM (1992). Atlas of the world.

[65] United Nations Statistics Division. World meteorological organization. http://data.un.org/Data.aspx?q=Dry+Bulb+Temperature&d=CLINO&f=ElementCode%3a01. Accessed 6 June 2013

[66] National Oceanic and Atmospheric Administration. Geographic Information Systems. National Climate Data Center

[67] Indiana State Department of Health (2010) Indiana Natality Report—2010. Table 4. Number of live births by month: Indiana residents, 2000–2010. In: Indiana State Department of Health, Epidemiology Research Center, Data Analysis Team. http://www.in.gov/isdh/reports/natality/2010/tb104.html. Accessed 11 May 2013

[68] Cyvin KB (1977). Seasonal variation of births dates of infants with unstable hips at birth. Acta Paediatr Scand.

[69] Eriksson AW, Lehmann W, Simpson NE, Milan FA (1980). Genetic studies on circumpolar populations. The human biology of circumpolar populations.

[70] Society for Applied Anthropology in Manitoba (2001). Summary of SAAM presentations. Anthropol Pract.

[71] Mellbin T (1962). The children of Swedish nomad Lapps. VII. Congenital malformations. Acta Orthop Scand.

[72] Getz B (1955). The hip joint in Lapps and its bearing on the problem of congenital dislocation. Acta Orthop Scand.

[73] Holck P (1991). The occurrence of hip joint dislocation in early Lappic populations of Norway. Int J Osteoarchaeol.

[74] Fredensborg N, Udén A (1976). Altered connective tissue in children with congenital dislocation of the hip. Arch Dis Child.

[75] Jensen BA, Relmann I, Fredensborg N (1986). Collagen type III predominance in newborns with congenital dislocation of the hip. Acta Orthop Scand.

[76] Skirving AP, Sims TJ, Bailey AJ (1984). Congenital dislocation of the hip: a possible inborn error of collagen metabolism. J Inherit Metab Dis.

[77] Sarban S, Baba F, Kocabey Y, Cengiz M, Isikan UE (2007). Free nerve endings and morphological features of the ligamentum capitis femoris in developmental dysplasia of the hip. J Pediatr Orthop B.

[78] Oda H, Igarashi M, Hayashi Y, Karube S, Inoue S, Sakaguchi R (1984). Soft tissue collagen in congenital dislocation of the hip—biochemical studies of the ligamentum teres of the femur and hip joint capsule. J Jpn Orthop Assoc.

[79] Carr AJ, Jefferson RJ, Benson MKDA (1993). Joint laxity and hip rotation in normal children and in those with congenital dislocation of the hip. J Bone Jt Surg [Br].

[80] Carter CO, Wilkinson JA (1964). Genetic and environmental factors in the etiology of congenital dislocation of the hip. Clin Orthop.

[81] Wynne-Davies R (1970). Acetabular dysplasia and familial joint laxity: two etiological factors in congenital dislocation of the hip. J Bone Jt Surg [Br].

[82] Carter C, Wilkinson J (1964). Persistent joint laxity and congenital dislocation of the hip. J Bone Jt Surg [Br].

[83] Fredensborg N (1976). Observations in children with congenital dislocation of the hp. Acta Orthop Scand.

[84] Czéizel A, Tusnády G, Vaczó G, Vozkelety T (1975). The mechanism of genetic predisposition in congenital dislocation of the hip. J Med Genet.

[85] Üden A, Lindhagen T (1988). Inguinal hernia in patients with congenital dislocation of the hip. A sign of general connective tissue disorder. Acta Orthop Scand.

[86] Eddie LW, Sutton B, Fitzgerald S, Bell RJ, Johnston PD, Tregear GW (1989). Relaxin in paired samples of serum and milk from women after term and preterm delivery. Am J Obstet Gynecol.

[87] MacLennan AH, Nicolson R, Green RC, Bath M (1986). Serum relaxin and pelvic pain of pregnancy. Lancet.

[88] Saugstad LF (1991). Persistent pelvic pain and pelvic joint instability. Eur J Obstet Gynecol Reprod Biol.

[89] Hinderaker T, Daltveit AK, Ingens LM, Udén A, Reikerås O (1994). The impact of intra-uterine factors on neonatal hip instability. Acta Orthop Scand.

[90] MacLennan AH, MacLennan SC (1997). Symptom-giving pelvic girdle relaxation of pregnancy, postnatal pelvic joint syndrome and developmental dysplasia of the hip. Acta Obstet Gynecol Scand.

[91] Forst J, Forst C, Forst R, Heller K-D (1997). Pathogenetic relevance of the pregnancy hormone relaxin to inborn hip instability. Arch Orthop Trauma Surg.

[92] Vogel I, Andersson JE, Uldjberg N (1998). Serum relaxin in the newborn is not a marker of neonatal hip instability. J Pediatr Orthop.

[93] Andrén L, Borglin NE (1961). A disorder of oestrogen metabolism as a causal factor of congenital dislocation of the hip. Acta Orthop Scand.

[94] Andrén L, Borglin NE (1961). Disturbed urinary excretion pattern of oestrogens in newborns with congenital dislocation of the hip. I. The excretion of oestrogen during the first few days of life. Acta Endocrinol.

[95] Andrén L, Borglin NE (1961). Disturbed urinary excretion pattern of oestrogens in newborns with congenital dislocation of the hip. II. The excretion of exogenous oestradiol-17β. Acta Endocrinol.

[96] Smith WS, Lieberg O, Goebelsmann U (1972). Estrogen determination in a pregnant wome with a family history of congenital dislocation of the hip. Clin Orthop.

[97] Kapoor B, Dunlop C, Wynn-Jones C, Fryer AA, Strange RC, Maffulli N (2007). Vitamin D and oestrogen receptor polymorphisms in developmental dysplasia of the hip and primary protrusio acetabuli—a preliminary report. J Negat Results Biomed.

[98] Desteli EE, Pişkin A, Gülman AB, Kaymaz F, Köksal B, Erdoğan M (2013). Estrogen receptors in hip joint capsule and ligamentum capitis femoris of babies with developmental dysplasia of the hip. Acta Orthop Traumatol Turc.

[99] Wojtys EM, Huston LJ, Boynton MD, Spindler KP, Lindenfeld TN (2002). The effect of the menstrual cycle on anterior cruciate ligament injuries in women as determined by hormone levels. Am J Sports Med.

[100] Garde AH, Hansen ÅM, Skovgaard LT, Christensen JM (2000). Seasonal and biological variation of blood concentrations of total cholesterol, dehydroepiandrosterone sulfate, hemoglobin A1 C, IgA, prolactin, and free testosterone in healthy women. Clin Chem.

[101] Ruyahel Y, Malm G, Haugen TB, Henrichsen T, Bjørsvik C, Grotmol T (2007). Seasonal variation in serum concentrations of reproductive hormones and urinary excretion of 6-sulfatoxymelatonin in men living north and south of the Arctic Circle: a longitudinal study. Clin Endocrinol.

[102] Aubertin-Leheudre M, Gorbach S, Woods M, Dwyer JT, Goldin B, Adlecruetz H (2008). Fat/fiber intakes and sex hormones in healthy premenopausal women in USA. J Steroid Biochem Mol Biol.

[103] Goldin BL, Adlercreutz H, Gorbach SL, Warram JH, Dwyer JT, Swenson L (1982). Estrogen excretion patterns and plasma levels in vegetarian and omnivorous women. NEJM.

[104] Adlercruetz H, Fotsis T, Bannwart C, Hämäkäinen E, Bloigu S, Ollus A (1986). Urinary estrogen profile determination in young Finnish vegetarian and omnivorous women. J Steroid Biochem.

[105] Katz Z, Lancet M, Skornik J, Chemke J, Mogilner BM, Klinberg M (1985). Teratogenicity of progestogens given during the first trimester of pregnancy. Obstet Gynecol.

[106] Yie S-M, Brown GM, Liu G-Y, Collins JA, Daya S, Hughes EG (1995). Melatonin and steroids in human pre-ovulatory follicular fluid: seasonal variations and granulosa cell steroid production. Hum Reprod.

[107] Combs GF (2012). The vitamins.

[108] Stryd RP, Gilbertson TJ, Brunden MN (1979). A seasonal variation study of 25-hydroxyvitamin D3 serum levels in normal humans. J Clin Endocrinol Metab.

[109] McKenna MJ (1992). Differences in vitamin D status between countries in young adults and the elderly. Am J Med.

[110] Harris SS, Dawson-Hughes B (1998). Seasonal changes in plasma 25-hydroxyvitamin D concentrations of young American black and white women. Am J Clin Nutr.

[111] Andersen R, Mølgard C, Skovgaard LT, Brot C, Cashman KD, Chabros E (2005). Teenage girls and elderly women living in northern Europe have low winter vitamin D status. Eur J Clin Nutr.

[112] Karohl C, Su S, Kumari M, Tangpricha V, Veledar E, Vaccarino V (2010). Heritability and seasonal variability of vitamin D concentrations in male twins. Am J Clin Nutr.

[113] Snellman G, Melhus H, Gedegorg R, Olofsson S, Wolk A, Pedersen NL (2009). Seasonal genetic influence on serum 25-hydorxyvitamin D levels: a twin study. PLoS One.

[114] Wacker M, Holick MF (2013). Vitamin D—effects on skeletal and extraskeletal health and the need for supplementation. Nutrients.

[115] Knight JA, Wong J, Blackmoer KM, Raboud JM, Vieth R (2010). Vitamin D association with estradiol and progesterone in young women. Cancer Causes Control.

[116] Aghajafari F, Ganulesapillai T, Ronskey PE, Tough SC, O’Beirne M, Rabi DM (2013). Association between maternal serum 25-hydroxyvitamin D level and pregnancy and neonatal outcomes: systematic review and meta-analysis of observational studies. BMJ.

[117] Christesen HT, Elvander C, Lamont RF, Jørgensen JS (2013). The impact of vitamin D in pregnancy on extraskeletal health in children: a systematic review. Acta Obstet Gynecol Scand.

[118] Paterson D (1976). The early diagnosis and treatment of congenital dislocation of the hip. Aust N Z J Surg.

[119] Lapunzina P, López Camelo JS, Rittler M, Castilla EE (2002). Risks of congenital anomalies in large for gestational age infants. J Pediatr.

[120] von Deimling U, Brähler JM, Niesen M, Wagner UA, Walpert J (1998). Der einfluß des geburtsgewichts auf die hüftreifung des neugeborenen (Effect of birth weight on hip maturation in the newborn infant). Klin Pädiatr.

[121] Holen KJ, Tegnander A, Tergesen T, Johansen OJ, Eik-Nes SH (1996). Ultrasonographic evaluation of breech presentation as a risk factor for hip dysplasia. Acta Paediatr.

[122] Hagenau T, Vest R, Gissel TN, Poulsen CS, Erlandsen M, Mosekilde L (2009). Global vitamin D levels in relation to age, gender, skin pigmentation and latitude: an ecologic meta-regression analysis. Osteoporos Int.

[123] Lucas RM, Ponsonby A-L, Dear K, Valery PC, Taylor B, van der Mei I (2013). Vitamin D status: multifactorial contribution of environment, genes and other factors in healthy Australian adults across a latitude gradient. J Steroid Biochem Mol Biol.

[124] Hedlund L, Brembeck P, Olausson H (2013). Determinants of vitamin D status in fair-skinned women of childbearing age at northern latitudes. PLoS One.

[125] Arguelles LM, Langman CB, Ariza AJ, Ali FN, Dilley K, Price H (2009). Heritability and environmental factors affecting vitamin D status in rural Chinese adolescent twins. J Clin Endocrinol Metab.

[126] Fernández-Alonso A, Valdera-Simbron J, Fiol-Ruiz G, Rodríguez-Sánchez F, Chedraui P, Pérez-López R (2011). First trimester serum levels of 25-hydroxyvitamind D, free β -human chorionic gonadotropin, and pregnancy-associated plasma protein A in Spanish women. Gynecol Endocrinol.

[127] Yoshimura N, Muraki S, Oka H, Morita M, Yamada H, Tanaka S (2013). Profiles of vitamin D insufficiency and deficiency in Japanese men and women: association with biological, environmental, and nutritional factors an coexisting disorders: the ROAD study. Osteoporos Int.

[128] Xiang J, Nagaya T, Huang X-E, Kuriki K, Imaeda N, Tokudome Y (2008). Sex and seasonal variations of plasma retinol, α-tocopherol, and carotenoid concentrations in Japanese dietitians. Asian Pac J Cancer Prev.

[129] Olmedilla B, Granado F, Blanco I, Rojas-Hidalgo E (1994). Seasonal and sex-related variations in six serum carotenoids, retinol, and α-tocopherol. Am J Clin Nutr.

[130] Faure H, Preziosi P, Roussel A-M, Bertrais S, Galan P, Hercberg S (2006). Factors influencing blood concentration of retinol, α-tocopherol, vitamin C, and β-carotene in the French participants of the SU.VI.MAX trial. Eur J Clin Nutr.

[131] Sivan Y, Laudon M, Tauman R, Zisapel N (2001). Melatonin production in healthy infants: evidence for seasonal variation. Pediatr Res.

[132] Kennaway DJ, Royles P (1986). Circadian rhythms of 6-sulphatoxy melatonin, cortisol and electrolyte excretion at the summer and winter solstices in normal men and women. Acta Endocrinol (Cph).

[133] Cos S, González A, Martínez-Campa C, Mediavilla C, Alonso-González C, Sánchez-Barceló EJ (2008). Melatonin as a selective estrogen enzyme modulator. Curr Cancer Drug Targets.

[134] Barron ML (2007). Light exposure, melatonin secretion, and menstrual cycle parameters: an integrative review. Biol Res Nurs.

[135] Morens DM, Pallansch MA, Rotbart HA (1995). Epidemiology. Human enterovirus infections.

[136] Sedmak G, Bina D, MacDonald J (2003). Assessment of an enterovirus sewage surveillance system by comparison of clinical isolates with sewage isolated from Milwaukee, Wisconsin, collected August 1994 to December 2002. Appl Environ Microbiol.

[137] Nagura S (1959). Zur frage der geographischen verbreitung der angeborenen hüftverenkung (On the problem of geographical distribution of congenital hip dislocation). Arch Orthop Unfallchir.

